# Magnetic Resonance Image Sequence Influences the Relationship between Bone Marrow Lesions Volume and Pain: Data from the Osteoarthritis Initiative

**DOI:** 10.1155/2015/731903

**Published:** 2015-11-02

**Authors:** Ming Zhang, Jeffrey B. Driban, Lori Lyn Price, Grace H. Lo, Timothy E. McAlindon

**Affiliations:** ^1^Division of Rheumatology, Tufts Medical Center, 800 Washington Street, Box No. 406, Boston, MA 02111, USA; ^2^The Institute for Clinical Research and Health Policy Studies, Tufts Medical Center and Tufts Clinical and Translational Science Institute, Tufts University, 800 Washington Street, Box No. 63, Boston, MA 02111, USA; ^3^Medical Care Line and Research Care Line, Houston VA HSR&D Center for Innovations in Quality, Effectiveness and Safety, Michael E. DeBakey VAMC, Houston, TX 77030, USA; ^4^Section of Immunology, Allergy, and Rheumatology, Baylor College of Medicine, 1 Baylor Plaza, BCM-285, Houston, TX 77030, USA

## Abstract

Subchondral bone marrow lesions (BMLs) are related to structural and symptomatic osteoarthritis progression. However, it is unclear how sequence selection influences a quantitative BML measurement and its construct validity. We compared quantitative assessment of BMLs on intermediate-weighted fat suppressed (IW FS) turbo spin echo and 3-dimensional dual echo steady state (3D DESS) sequences. We used a customized software to measure 30 knees' (24- and 48-month MR images) BMLs on both sequences. The results showed that the IW FS sequences have much larger BML volumes (median: IW FS = 1840 mm^3^; DESS = 191 mm^3^) and BML volume change (between 24 and 48 months) than DESS sequence and demonstrate more BML volume change. The 24-month BML volume on IW FS is correlated with BML volume on DESS (*r*_*s*_ = 0.83). BML volume change on IW FS is not significantly correlated with change on DESS. The 24-month WOMAC pain is correlated with the 24-month BMLs on IW FS (*r*_*s*_ = 0.39) but not DESS. The change in WOMAC pain is correlated with BML volume change on IW FS (*r*_*s*_ = 0.37) but not DESS. Overall, BML quantification on IW FS offers better validity and statistical power than BML quantification on a 3D DESS sequence.

## 1. Introduction

Subchondral bone marrow lesions (BMLs) are common findings on magnetic resonance (MR) images of knees with osteoarthritis (OA) and relate to structural and symptomatic progression of OA [1–3]. While BMLs are often assessed on intermediate-weighted fat suppressed (IW FS) or similar sequences [4] some researchers have also evaluated BMLs on 3-dimensional dual echo steady state (3D DESS) sequences or other similar sequences that are used for cartilage measurements [5]. The latter approach enables a time- and cost-efficient method to assess changes in BMLs and cartilage on the same sequence [6]. One prior study provided a head-to-head cross-sectional comparison using a semiquantitative measure of BML and demonstrated that IW FS sequences are more sensitive to detecting BMLs [7]. No studies to our knowledge have evaluated the measures longitudinally nor have any studies compared the association of BMLs measured with the two different sequences as they relate with pain, which would provide insight into the construct validity of the BMLs measured using the two different sequences [7]. The purpose of this study was to compare assessment of BMLs on IW FS and 3D DESS sequences both cross-sectionally and longitudinally using quantitative assessments and to evaluate their construct validity against knee pain. We anticipated that BML volumes and change of volumes would be larger on IW FS than on a 3D DESS sequence but that construct validity would be similar.

## 2. Methods

### 2.1. Participants

We chose 30 knees from the Osteoarthritis Initiative (OAI) with 24- and 48-month MR images as well as complete data from the OAI Bone Ancillary Study (i.e., subchondral bone mineral density, MR-based trabecular morphometry, meniscal readings, and cartilage damage). We enriched the study sample by selecting knees with or without medial joint space narrowing to increase the heterogeneity of BML size and BML change. Fifteen knees were selected among those with an increase in medial joint space narrowing (OARSI score) between the 24- and 48-month OAI visits. An additional 15 knees with no increase in medial joint space narrowing were also selected. Increase in joint space narrowing was defined as any increase in OARSI joint space narrowing score [8] including within-grade changes.

### 2.2. MR Images

All of the knees had IW FS and 3D DESS MR images. The IW FS turbo spin echo and 3D DESS sequences were acquired using the OAI MR imaging protocol [9]. The IW FS sequence with field of view = 160 mm, slice thickness = 3 mm, skip = 0 mm, flip angle = 180 degrees, echo time = 30 ms, recovery time = 3200 ms, 313 × 448 matrix, *x* resolution = 0.357 mm, *y* resolution = 0.511 mm, and total slice number = 37. The 3D DESS sequence with field of view = 140 mm, slice thickness = 0.7 mm, skip = 0 mm, flip angle = 25 degrees, echo time = 4.7 ms, recovery time = 16.3 ms, 307 × 384 matrix, *x* resolution = 0.365 mm, *y* resolution = 0.456 mm, and total slice number = 160.

### 2.3. Semiautomated BML Segmentation

We designed a customized semiautomatic software to measure BMLs on both sequences. “BMLs are characterized as areas of high-signal intensity within bone on fat suppressed MR images [10, 11]. One reader used the software to place a large region of interest (ROI) around a BML. The software first applies threshold filter to convert selected ROI into binary image. The threshold is calculated based on the intensity histogram distribution within the region of interest. The follow-up slice used the same threshold on the corresponding baseline slice. Then a dilation filter is used to merge connected regions. Finally, the software removes small noise pixels. The user performs a final quality control to ensure the BMLs had been correctly segmented on each slice. The user can manually adjust the threshold and remove non-BML regions. To colocate the corresponding BMLs on baseline and follow-up images, we used dual screens to display simultaneously baseline and follow-up MR images.” We summed the femur and tibia BMLs to generate a whole knee BML volume.

We first measured 30 pairs (baseline and follow-up) of IW FS images and then 30 pairs of 3D DESS images. We randomly selected 15 knees from the analytic dataset to assess intratester reliability. The two measurements were separated by at least 72 hours. Intratester (ICC [3, 1 model] [12]) reliability for IW FS baseline is 0.99 and IW FS change is 0.84; the reliability for 3D DESS baseline is 0.97 and 3D DESS change is 0.93.

### 2.4. Clinical Data

Knee pain was measured using the Western Ontario and McMaster University (WOMAC) pain score [13]. Radiographic measure of joint space narrowing (JSN) has been previously described in detail [14]. The radiographs, central readings, and protocols are publicly available at the OAI website (kxr_sq_bu_00 [version 0.5] and kxr_sq_bu_03 [version 3.5]; http://oai.epi-ucsf.org/; reliability for these readings was good with kappa = 0.70 to 0.88.)

### 2.5. Statistical Analyses

An* a priori* power calculation revealed 30 participants were needed to detect a Pearson correlation of 0.50 with *α* = 0.05 and 80% power. A post hoc power calculation showed that, using the same parameters, we have >75% power to detect a Spearman correlation >0.50. To determine the distribution of whole knee BML volumes on each sequence we calculated medians and the 25th, 75th percentiles for BML volumes on both sequences. We performed a Wilcoxon signed rank sum test to compare BML volumes on the IW FS and 3D DESS sequences. Bland-Altman-like plots [15] were generated using the median (rather than the mean) of the difference for the horizontal line. We calculated Spearman correlations (*r*_*s*_) to assess the relationship of 24-month BML volume and BML volume change (48-month BMLs minus 24-month BMLs) between IW FS and 3D DESS sequences. Finally, to determine construct validity we calculated Spearman correlations between 24-month BMLs on both sequences with 24-month WOMAC pain score. We also calculated the Spearman correlations of BML volume change on both sequences with WOMAC pain change (48-month WOMAC pain score minus 24-month WOMAC pain score).

## 3. Results

The study sample consisted of 30 right knees among 16 males and 14 females. The mean age was 64.0 (SD 9.4) years and mean body mass index was 30.7 (SD 5.3) kg/m^2^, with a mean WOMAC pain score of 4.4 (3.9) and an average change in WOMAC pain of 0.7 (3.7) over 24 months of follow-up. Twenty-five knees (83%) had radiographic OA (Kellgren-Lawrence grade ≥ 2).

### 3.1. BMLs on IW FS and 3D DESS Sequences

**Figure 1:**
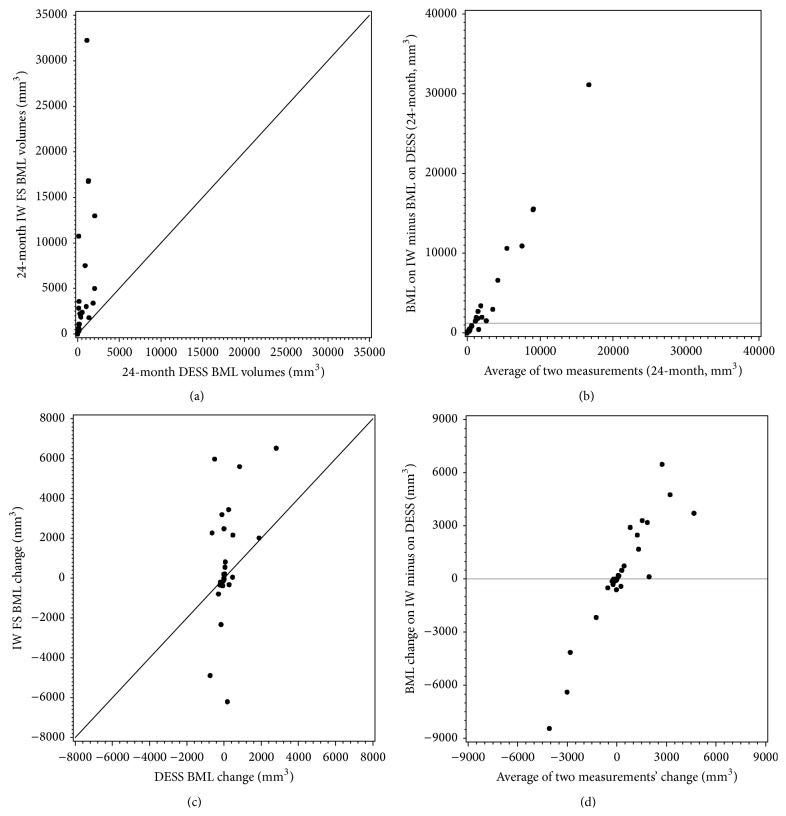
(a) Scatter plot of baseline BML volumes on IW FS versus 3D DESS. (b) Modified Bland-Altman plot of IW FS minus 3D DESS. (c) Scatter plot of BML volume change on IW FS versus 3D DESS. (d) Modified Bland-Altman plot of BML volume change on IW FS minus change on 3D DESS.

There were 87 BMLs on IW FS 24-month visit. 3D DESS detected 75% of them (65 out of 87 BMLs). BMLs measured on the IW FS sequences had statistically significantly larger volumes than those measured on 3D DESS sequences (*p* < 0.0001, 24-month BMLs IW FS = 1840 (median) [290,3588] (25th and 75th percentiles) mm^3^; 3D DESS = 191 [40,1048] mm^3^; Figure 1(a)). The difference in BML volumes between sequences is greater among knees with larger BMLs than knees with smaller BMLs (the larger the BMLs, the greater the difference between sequences, Figure 1(b)).

The IW FS sequence generally demonstrated more BML volume change (BMLs change between 24-month and 48-month: IW FS = 27 [−320, 2166] mm^3^; DESS = 2 [−110, 187] mm^3^) than 3D DESS sequence (Figure 1(c)). The difference of BML volume change between the two sequences was greatest among knees with larger BML volume change (Figure 1(d)).

The 24-month BML volume on IW FS was correlated with the 24-month BML volume on 3D DESS (Figure 1(a), *r*_*s*_ = 0.83, 95% confidence interval [95% CI] = 0.66 to 0.91). However, BML volume change on IW FS was not significantly correlated with the BML volume change on 3D DESS (Figure 1(c), *r*_*s*_ = 0.33, 95% CI = −0.04 to 0.61).

Three knees did not have any BML volume detected by either sequence at either time point. IW FS detected a larger absolute change than DESS in 26 of the 27 knees that had BMLs.

### 3.2. BMLs and WOMAC Pain

The 24-month WOMAC pain was statistically significantly correlated with the 24-month BML volume on IW FS (*r*_*s*_ = 0.39, 95% CI = 0.02 to 0.65; see Supplemental Figure  1(A) in Supplementary Material available online at http://dx.doi.org/10.1155/2015/731903) but not the 24-month BML volume on 3D DESS (*r*_*s*_ = 0.27, 95% CI = −0.11 to 0.57; see Supplemental Figure  1(B)). The change in WOMAC pain was correlated with BML volume change on IW FS (*r*_*s*_ = 0.37, 95% CI = 0.01 to 0.64) but not with BML volume change on 3D DESS (*r*_*s*_ = −0.19, 95% CI = −0.51 to 0.19).

## 4. Discussion

This study confirms that the selection of appropriate MR pulse sequence to measure BMLs is important. We verified our hypothesis that IW FS sequences are more sensitive in detecting BMLs as compared to DESS sequences, sequences that are optimized to evaluate articular cartilage, as expected. We also found that the correlation between BML volume and knee pain was* qualitatively* greater in magnitude and statistically significant when using BML measurements from the IW FS sequences compared to those measured using DESS sequences. Although evaluating BMLs on 3D DESS sequences would enable a time- and cost-efficient method to assess changes in BMLs and cartilage on the same sequences, our results indicate that a study doing so may require a larger sample size to overcome the lack of sensitivity for measuring BMLs, especially those of larger volume, and decreased correlations with pain.

**Figure 2:**
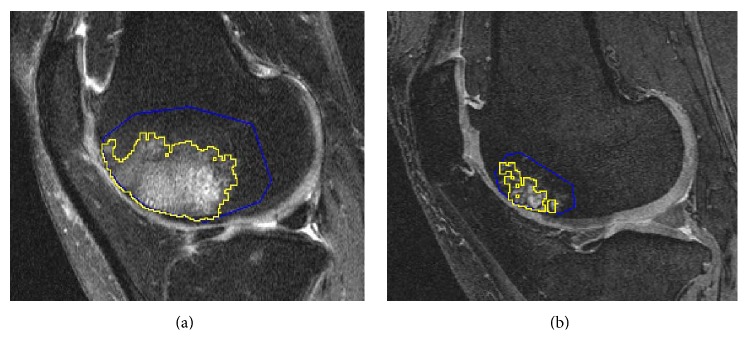
(a) BML on IW FS sequence. (b) Same BML on 3D DESS sequence.

In a recent published paper [16], there were 74% BMLs on T2-weighted sequences which were also seen on T1-weighted sequences, similar to our results (75% BMLs on IW FS sequence were detected on 3D DESS sequence). We also found that the sizes of BML measured on IW FS generally are larger than those measured on 3D DESS (Figure 2). The cross-sectional study by Hayashi et al., using a semiquantitative BML assessment, similarly found that measurements taken using IW FS sequences demonstrate larger subchondral BMLs in 186 (93%) subregions when compared to the DESS sequences [7].

BMLs are an important feature of knee OA that is associated with pain [1, 17, 18]. In this study, we found BML volume and BML volume change on IW FS sequence had stronger associations with knee pain and knee pain fluctuation than when BMLs were measured on 3D DESS sequences.

An important limitation of this study is its small sample size. However, by selecting a small sample size, this allowed us to detect differences in the strength of associations between both cross-sectional and longitudinal change in BML volumes measured using the two sequences as compared with WOMAC pain.

## 5. Conclusions

Generally, BMLs are detected on both IW FS and 3D DESS sequences. There is an association of BML volumes on both sequences at baseline though the point estimates are smaller when assessing BML volume change. IW FS sequence usually has larger BML volumes than DESS sequence and may be more sensitive to change. The quantitative BMLs measurement on IW FS sequence also provided larger correlation coefficients with pain than the 3D DESS both cross-sectionally and longitudinally. Overall, these results do not support the use of DESS sequences as optimal sequence to measure BMLs. While it is feasible, BMLs measured on 3D DESS sequences will underestimate BML size, BML change, and some associations (e.g., with knee pain).

## Supplementary Material

Supplementary figures are the plots for BML and WOMAC pain using ranks: Plots for BML and WOMAC pain using ranks. A. Scatter plot of knee pain (knees ranked by severity) and knees ranked according to BML volume on intermediate weighted, fat suppressed (IWFS) images. B. Scatter plot of knee pain (knees ranked by severity) and knees ranked according to BML volume on Dual Echo Steady State (DESS) images.
