# Supplementary material for: Magnetic Resonance Image Sequence Influences the Relationship between Bone Marrow Lesions Volume and Pain: Data from the Osteoarthritis Initiative
Source: Biomed Res Int. 2015 Nov 2;2015:731903. doi: 10.1155/2015/731903 (PMC4644821; doi:10.1155/2015/731903)
Supplement: Supplementary file 1 — Supplementary figures are the plots for BML and WOMAC pain using ranks: Plots for BML and WOMAC pain using ranks. A. Scatter plot of knee pain (knees ranked by severity) and knees ranked according to BML volume on intermediate weighted, fat suppressed (IWFS) images. B. Scatter plot of knee pain (knees ranked by severity) and knees ranked according to BML volume on Dual Echo Steady State (DESS) images. [file 731903.f1.pdf]

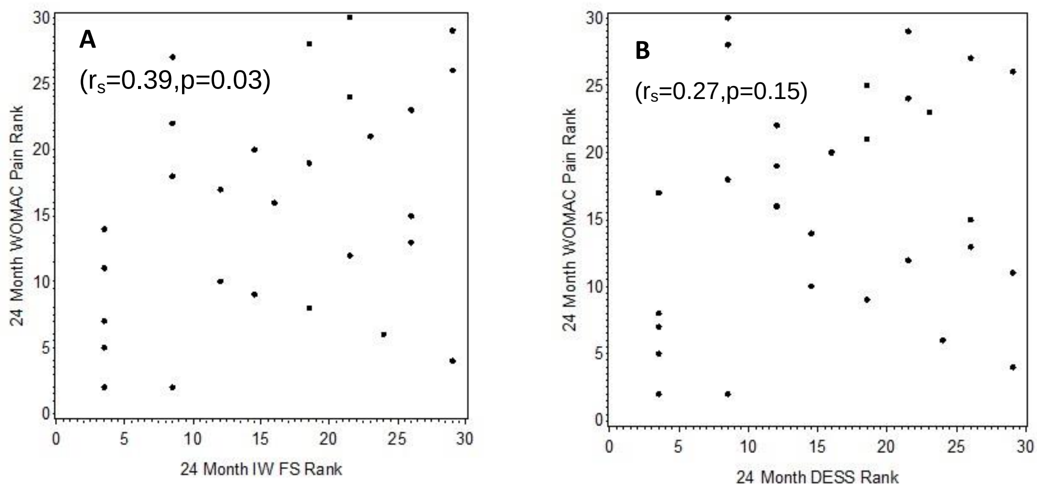

Supplemental Figure 1. Plots for BML and WOMAC pain using ranks. A. Scatter plot of knee pain (knees ranked by severity) and knees ranked according to BML volume on intermediate weighted, fat suppressed (IWFS) images. B. Scatter plot of knee pain (knees ranked by severity) and knees ranked according to BML volume on Dual Echo Steady State (DESS) images.
